# LasΔ5315 Effector Induces Extreme Starch Accumulation and Chlorosis as *Ca*. Liberibacter asiaticus Infection in *Nicotiana benthamiana*

**DOI:** 10.3389/fpls.2018.00113

**Published:** 2018-02-07

**Authors:** Marco Pitino, Victoria Allen, Yongping Duan

**Affiliations:** US Horticultural Research Laboratory, USDA-ARS, Fort Pierce, FL, United States

**Keywords:** citrus huanglongbing, liberibacter asiaticus, effector, starch, *nicotiana benthamiana*

## Abstract

Huanglongbing (HLB), a destructive plant bacterial disease, severely impedes worldwide citrus production. HLB is associated with a phloem-limited α-proteobacterium, *Candidatus* Liberibacter asiaticus (Las). Las infection causes yellow shoots and blotchy mottle on leaves and is associated with excessive starch accumulation. However, the mechanisms underlying the starch accumulation remain unknown. We previously showed that the Las5315mp effector induced callose deposition and cell death in *Nicotiana benthamiana*. In this study, we demonstrated that Las can experimentally infect *N. benthamiana* via dodder transmission. Furthermore, we revealed another key function of the Las5315 effector by demonstrating that transient expression of the truncated form of the effector, LasΔ5315, induced excessive starch accumulation by 6 fold after 8 dpi in *N. benthamiana* after removal of the chloroplast transit peptide from the Las5315mp. The induction mechanisms of LasΔ5315 in *N. benthamiana* were attributed to the up-regulation of ADP-glucose pyrophosphorylase, granule-bound starch synthase, soluble starch synthase, and starch branching enzyme for increasing starch production, and to the significant down-regulation of the starch degradation enzymes: alpha-glucosidase, alpha-amylase, and glycosyl hydrolase for decreasing starch degradation. This is the first report that Las can infect the model plant *N. benthamiana*. Using this model plant, we demonstrated that the LasΔ5315 effector caused the most prominent HLB symptoms, starch accumulation and chlorosis as Las infection in *N. benthamiana*. Altogether the Las 5315 effector is critical for Las pathogenesis, and therefore, an important target for interference.

## Introduction

Huanglongbing (HLB) is one of the most complex and severe diseases that affects all commercial citrus species. HLB is associated with an unculturable phloem-limited α-proteobacterium *Candidatus* Liberibacter (Garnier and Bové, 1983; Jagoueix et al., 1994) and is transmitted by two species of citrus psyllids, *Diaphorina citri* Kuwayama (Asian citrus psyllid: ACP) and *Trioza erytreae* (del Guercio) (African citrus psyllid) (Bove, 2006). The most noticeable symptoms of HLB are yellow shoots and blotchy mottle, a random pattern of yellowing on leaves. HLB infection eventually renders infected plants completely unproductive and causes death within 3–5 years. Another common HLB characteristic is callose deposition and extremely high starch accumulation in leaves (Yelenosky and Guy, 1977), which is believed to result in disintegration of the chloroplast thylakoid system and thus the observed chlorosis in infected tissues (Schaffer et al., 1986). There is no known cure for HLB and no commercially viable, effective treatment.

*Ca*. Liberibacter asiaticus (Las) is a bacterial plant pathogen with a dual host life cycle dependent on sap-feeding insects for transmission to plants. Pathogens may also manipulate the development of their psyllid hosts to improve growth, enhance colonization, and increase transmission (Sugio et al., 2011; Win et al., 2012; Petre et al., 2014). Protein secretion is a key virulence mechanism of pathogenic and symbiotic bacteria, and the presence of genes coding for a complete Sec-translocon in Las (Duan et al., 2009) suggests an important role for the secretion of proteins including virulence factors. Moreover, Las seems to possess effectors with the ability to manipulate plant physiology (Pitino et al., 2016).

We previously identified 16 Las candidate effector proteins (Pitino et al., 2016) using a fast-forward screen pipeline for Las candidate effectors containing signal peptide (SP), which is usually required to achieve final folding and localization of exported proteins outside the prokaryotic cell (Pugsley, 1993; Tuteja, 2005; Jehl et al., 2011; Palmer and Berks, 2012). We found that one particular effector (Las5315) caused cell death when transiently expressed in *Nicotiana benthamiana* without the SP which is normally cleaved by signal peptidases (Las5315mp). This effector induces callose deposition and targets the chloroplast. Interestingly, the excessive callose formation in the phloem seems to be linked to a plant defense reaction to Las bacterium. Las infection processes can lead to perturbation of normal carbon partitioning, excessive starch accumulation in plastids, chloroplast disruption, increase of H_2_O_2_, insufficient root development, reduced size and aberrant fruit development, and slow growth (Bove, 2006; Albrecht and Bowman, 2008; Folimonova and Achor, 2010; Gottwald, 2010; Rosales and Burns, 2011; Koh et al., 2012; Aritua et al., 2013; Martinelli et al., 2013).

Starch can be classified into two types: transitory starch, which occurs in photosynthetic organs and is also called leaf starch, and reserve starch, which occurs in storage organs (Wang et al., 2013). The starch accumulated during the day is degraded during the subsequent night, providing a continued supply of carbohydrates in the absence of photosynthesis (Gibon et al., 2009). In higher plants the production of starch is orchestrated by chloroplast-localized biosynthetic enzymes affected by endogenous hormone levels that regulate genes involved in the starch metabolism pathway (Liu et al., 2015). Viral factors can affect chloroplast ultrastructure, symptom development and starch accumulation (Allan et al., 2001; Zhao et al., 2016); for example, TMV movement protein induces starch accumulation in transgenic plants (Olesinski et al., 1996). In addition, exudates from fungal spores induced starch accumulation in wild-type *Lotus japonicus* roots (Gutjahr et al., 2009). *Sinorhizobium meliloti* flavodoxin-overexpressing rhizobia also led to high starch accumulation in nodules (Redondo et al., 2009). Leaves exposed to volatiles emitted by *Alternaria alternata* revealed that starch overaccumulation was accompanied by up-regulation of starch synthesis genes (Ezquer et al., 2010); emission of Microbial volatile organic compounds (MVOCs) strongly promoted starch accumulation in leaves of both mono-and dicotyledonous plants (Kanchiswamy et al., 2015). *Ca*. Liberibacter asiaticus, the presumptive causal agent of HLB, is another organism that induces excessive starch accumulation (Yelenosky and Guy, 1977), yet the mechanism remains unclear.

Excessive starch accumulation is observed in the phloem sieve elements of Las infected citrus (Etxeberria et al., 2009; Folimonova and Achor, 2010), which is not common in higher plants. In fact, starch induction by Las is so extreme that the starch content of citrus leaves can be used for diagnosis of HLB (Whitaker et al., 2014). Originally, starch accumulation was thought to result from necrotic phloem blockages which disrupt the natural carbon source/sink balance of the plant tissues (Schneider, 1968), however more recently, it has been shown that granule-bound starch synthase is up-regulated well before plants become symptomatic (Nwugo et al., 2013a). This apparent conflict underscores the need for further research into the host-pathogen dynamics of starch accumulation. Transcriptomic and proteomic studies have revealed that several enzymes related to starch synthesis, degradation, and transport are up or downregulated in response to Las infection (Martinelli et al., 2012; Nwugo et al., 2013a). These include large subunit ADP-glucose pyrophosphorylase (Kim et al., 2009; Martinelli et al., 2012; Aritua et al., 2013), various starch synthase isoforms, granule-bound starch synthase, glucanotransferase, maltose excess protein, beta-amylases, glucoamylase, and glycosyltransferase (Fan et al., 2010; Martinelli et al., 2012; Aritua et al., 2013; Nwugo et al., 2013a; Xu et al., 2015). Additionally, increased activity of cell-wall invertase, a protein involved in carbohydrate transport and linked to starch accumulation, has been observed in Las-infected leaves (Fan et al., 2010). The molecular mechanism by which Las infection induces these changes remains unknown.

In this study, we revealed how LasΔ5315 induced extreme starch accumulation and severe chlorosis, typical HLB symptoms as seen both in Las-infected *N. benthamiana* and citrus. We also confirmed *N. benthamiana* is an experimental host plant of *Ca*. Liberibacter asiaticus.

## Materials and methods

### LasΔ5315

Downstream of the N-terminal SP sequence of Las5315 effector protein, we previously found a chloroplast transit peptide domain 56 a.a. in length using ChloroP 1.1 (Pitino et al., 2016). In this work we generated a new construct with deletion of the chloroplast transit peptide, referred to as LasΔ5315. The sequence was prepared by amplification of corresponding fragments using polymerase chain reaction (PCR). Primers were tailed with attB1 and attB2 sites. The sequences were amplified using Las infected periwinkle DNA and HIFI PCR master mix (Clontech). The PCR fragments were first cloned into pDONR/Zeo by Gateway® BP Clonase® II (Invitrogen) and then subcloned into the Gateway destination vector ImpGWB405 (Nakagawa et al., 2007).

### Transient expression of *Las*Δ*5315* in *N. benthamiana* and localization

*Agrobacterium tumefaciens* GV3101 transformant cells carrying Las5315, Las5315mp, and LasΔ5315 in vector ImpGWB405 (Nakagawa et al., 2007) were cultured overnight in LB medium with 50 μg ml^−1^ of rifampicin and 100 μg ml^−1^ spectinomycin and resuspended in 10 mM MgCl_2_. The culture was diluted to an optical density at 600 nm of 0.5, and acetosyringone (final concentration of 100 μM) was added. For each construct, we infiltrated three leaves of three 4 week-old *N. benthamiana* plants with the *A. tumefaciens* suspension. The agro-infiltrated leaves were analyzed for protein localization at 3 dpi under a microscope (Olympus BX51-P) equipped with a UV light source. Agroinfiltrated plants were kept in a greenhouse for the duration of the experiment and observed for phenotypic changes.

### Starch staining and quantitation

Starch was visualized by iodine staining reagent (Sigma). Four dpi detached leaves or Las infected leaves were boiled in 95% ethanol to remove leaf pigments thoroughly and then washed twice with deionized water. Rehydrated leaves were stained for 10 min in 5% iodine solution (5% [w/v] I_2_ and 10% [w/v] KI) and incubated in water to allow fading until a clear background was obtained. Iodine staining was repeated at least three times for each construct.

Enzymatic measurement of starch in leaves was performed using Starch Fluorometric Assay Kit (BioVision, California, US), 1 leaf disc was collected using cork borer number 6 at 2, 4, 6, and 8 dpi from tissue agro-infiltrated with the corresponding constructs. Leaf discs were pulverized using liquid nitrogen and then starch was extracted and digested following the manufacturer's instructions. After the digestion the samples were measured at Ex/Em wavelength 535/587 nm by the LUMIstar microplate reader (BMG Labtech). Three biological replicates were used for the analyses.

### Protoplast extraction and starch iodine stain

Detached leaves were rinsed with distilled water and blotted dry. Leaves were sliced with a razor blade into 1- to 2-mm strips and placed in 20 ml of filter-sterilized Enzyme Solution (0.5 M sucrose, 10 mM MES-KOH [pH 5.7], 20 mM CaCl2, 40 mM KCl, 1% Cellulase R-10, 1% Macerozyme R-10). Plant tissue was shaken at 35 rpm in the dark at room temperature for 16–18 h. Protoplasts were collected by sieving through 8 layers of cheesecloth and centrifuged for 7 min at 100 g. Supernatant was removed, and the pellet was used for iodine staining and microscope analysis.

### Immunoblot detection of LasΔ5315 in *N. benthamiana*

Two leaf discs were collected from the infiltrated part of *N. benthamiana* leaves at 4 dpi using a 6 mm cork borer; leaf discs were ground in liquid nitrogen and suspended in extraction buffer plus protease inhibitor cocktail as per the manufacturer's protocol (denaturing). Proteins were separated by SDS-PAGE 4–12% acrylamide gel and electro-blotted to a nitrocellulose membrane using an iBlot blotting system (Life Technologies). The membrane was blocked with TBS-T containing 5% skimmed milk powder for 1 h at room temperature.

For immunoblot analysis, antibodies for LasΔ5315 were generated against a peptide sequence of LasΔ5315 (CISRTRIDSSPPPHG) (GenScript) and then HRP conjugated using Lighting-Link HRP kit (Innova Bioscience). The membrane was first incubated with primary antibody for 1 h with mild agitation and then washed 3 × 10 min; protein band was detected and analyzed using WestenSure Chemiluminescence substrate and the digital imaging system C-DiGit® Blot Scanner (LI-COR).

### Chlorophyll assay

Leaf tissues were inspected at 4 dpi for the development of localized chlorosis around the point of infiltration. Chlorophyll assays were carried out using leaf discs excised with a cork borer number 6 from three individual plants, two leaf discs per leaf, for a total of six leaf discs. Each sample with two leaf discs was incubated for 24 h in 2 mL 95% ethanol at 4°C in the dark. Chlorophyll concentrations were determined using chlorophyll equations based on the formula: 13.70 · A_665_–5.76 · A_649_ used − 7.60 · A_665_ + 25.8 · A_649_ (Ritchie, 2006).

### RNA extraction and cDNA synthesis

Total RNA was extracted from 3 separate agroinfiltrated leaves using 3 *N. benthamiana* plants at the end of the photoperiod (day) and immediately before onset of the photoperiod (night). Sample leaves were quickly frozen in liquid nitrogen and ground to a powder using an autoclaved mortar and pestle. Total RNA was extracted using TRIzol Reagent (Invitrogen). Total RNA concentration and purity were determined from the ratio of absorbance readings at 260 and 280 nm, using a Nanodrop 1000 spectrophotometer (Thermo Scientific). cDNA synthesis was performed with poly-T primers using the M-MLV reverse transcriptase system (Promega) according to the manufacturer's instructions.

### Gene expression analysis

Primers were purchased from IDT and used in a 15 μL reaction with 7.5 μL of 2 × FAST SYBR Green Master Mix (Quanta Bio) reagent and 2 μL of DNA template. The following standard thermal profile was used for all amplifications: 95°C for 5 min, followed by 40 cycles of 95°C for 3 s and 62°C for 30 s. Primer sequences are listed in Table S1. Reactions were performed in triplicate and normalized to EF1 expression; the 2^−ΔΔCt^ method was used to calculate relative expression as previously described (Pitino et al., 2015).

### Transmission procedure and genomic DNA extraction for Las detection

Dodder (*Cuscuta pentagona*) germinated on Las-infected Duncan citrus plants was used to inoculate *N. benthamiana* with Las bacteria. Connections were established on 4 week-old *N. benthamiana* plants and maintained for 4 weeks, after which the dodder was removed. Control plants were connected with uninfected dodder for the same time period. Plants were kept in a greenhouse.

To determine Las bacterial titer, symptomatic and healthy leaves were collected from *N. benthamiana* 1 and 2 and 3 months after dodder inoculation and tested using primers HLBasf, HLBr and probe HLBp targeting the 16S rDNA of Las (Li et al., 2006) as described previously (Pitino et al., 2017). Starch staining was also carried out on controls and infected *N. benthamiana* immediately before onset of the photoperiod (night) as described above.

## Results

### Expression of *Las*Δ*5315* results in a dramatic visual phenotype and accumulates in vescicle structures

**Figure 1:**
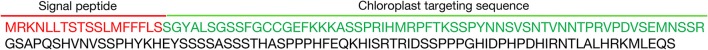
Sequence and expression of LasDelta5315 in *N. benthamiana* adapted from Pitino et al. (2016). Amino acid sequence of full-length Las5315. Signal peptide sequence shown in red; chloroplast transit peptide shown in green. Sequence of LasΔ5315 shown in black.

**Figure 2:**
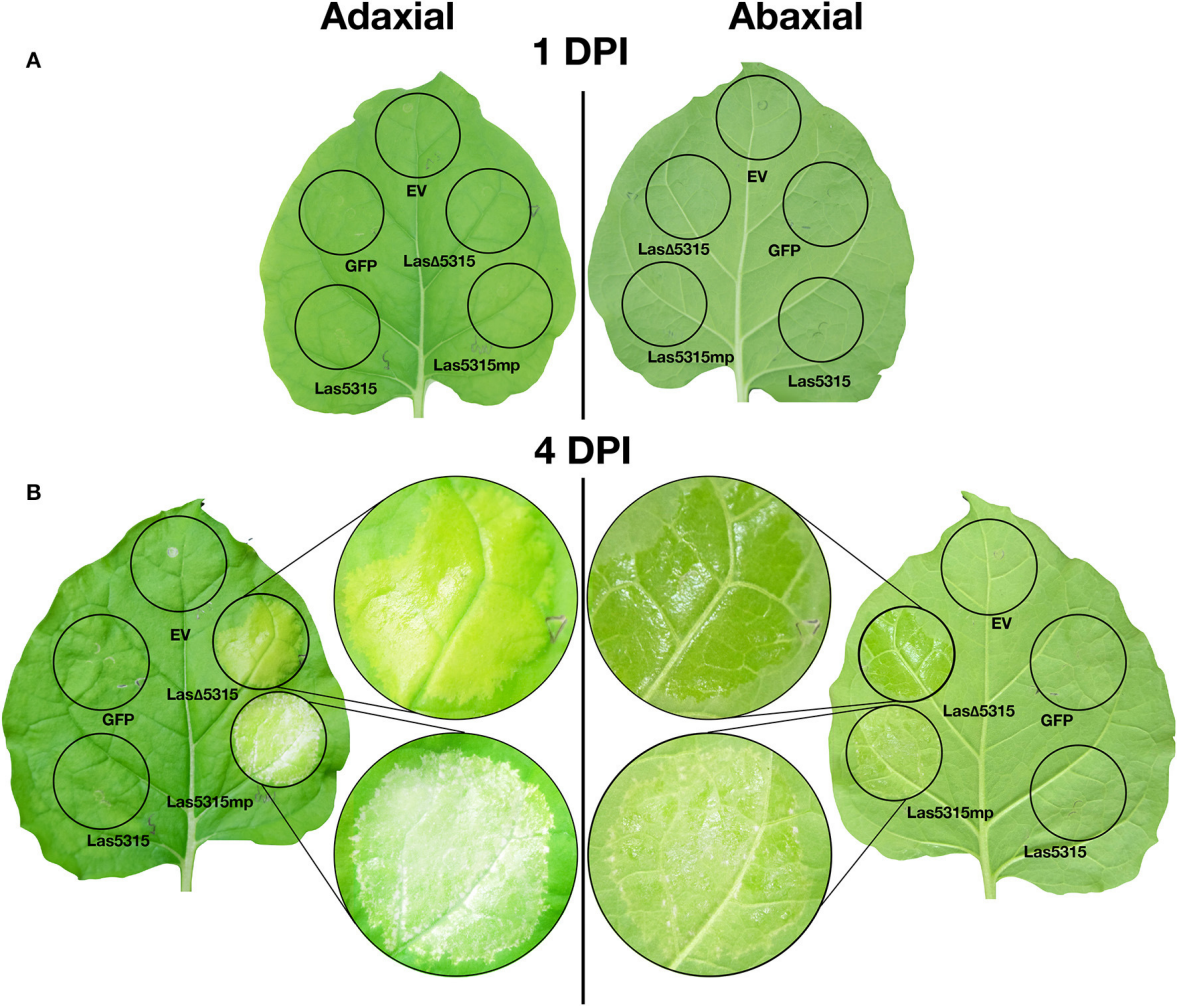
Visual phenotypes of leaves expressing Las5315, Las5315mp, LasΔ5315, GFP, and an empty vector. **(A)** At 1 day post infiltration (dpi), no visible phenotypes are apparent on the leaf. **(B)** At 4 DPI, the zone of infiltration where Las5315mp is expressed shows cell death. The zone of inhibition where LasΔ5315 is expressed shows a chlorotic, yellowing phenotype on the adaxial side and a water-soaking phenotype on the abaxial side. Zones of infiltration where Las5315, GFP, or an empty vector (EV) are expressed remain healthy and green.

The Las5315 effector is composed of a SP at the N-terminal, followed by a chloroplast transit peptide and the remainder of the protein (Figure 1). In our previous study we observed robust phenotypes upon expressing Las5315 with the SP deleted (Las5315mp). Like the secretory SP, it is likely that the chloroplast transit peptide is also cleaved *in vivo* once Las5315mp has localized to the chloroplast. Transit peptide directs post-translational localization and is removed upon arrival in the organelle (Bruce, 2000; Shen et al., 2017). Therefore, in order to further characterize Las5315, we created a construct lacking both the secretory peptide and the chloroplast transit peptide (LasΔ5315). *Las*Δ*5315* was transiently expressed in *N. benthamiana* using *Agrobacterium*-mediated expression. The protein was detectable by western blot 2 days post-infiltration (dpi) (Figure S1), yielding a single band with a molecular mass of slightly less than 37 kilodaltons (kDa). This was consistent with the expected combined molecular mass of LasΔ5315 (8 kDa) and GFP (27 kDa). Three leaves were agroinfiltrated with the constructs (Figure 2A), and 4 days after agroinfiltration, the treated areas exhibited chlorosis on the adaxial side of the leaf, and a noticeably water-soaking appearance on the abaxial side (Figure 2B, Figures S2A,S3A). In contrast, cell death was observed in the zone of infiltration with Las5315mp, as in our previous study (Pitino et al., 2016), (Figure 2B, Figure S3B). Agroinfiltration with a control vector containing only GFP did not cause any visible phenotype (Figure 2B, Figure S3C).

In order to establish the localization pattern of LasΔ5315, we imaged the leaf epidermal layer of the infiltrated zones and compared to zones infiltrated with the control GFP vector. While the control showed diffuse fluorescence (Figures S4A,B), GFP-tagged LasΔ5315 accumulated in small and large vescicles throughout the cytosol (Figures S4C–F). Interestingly, these vescicles often contained one or more chloroplasts, in which fluorescence was absent. This suggests that LasΔ5315 protein does not enter chloroplasts, but rather envelops them.

### Expression of *Las*Δ*5315* causes visible starch accumulation revealed by iodine staining

**Figure 3:**
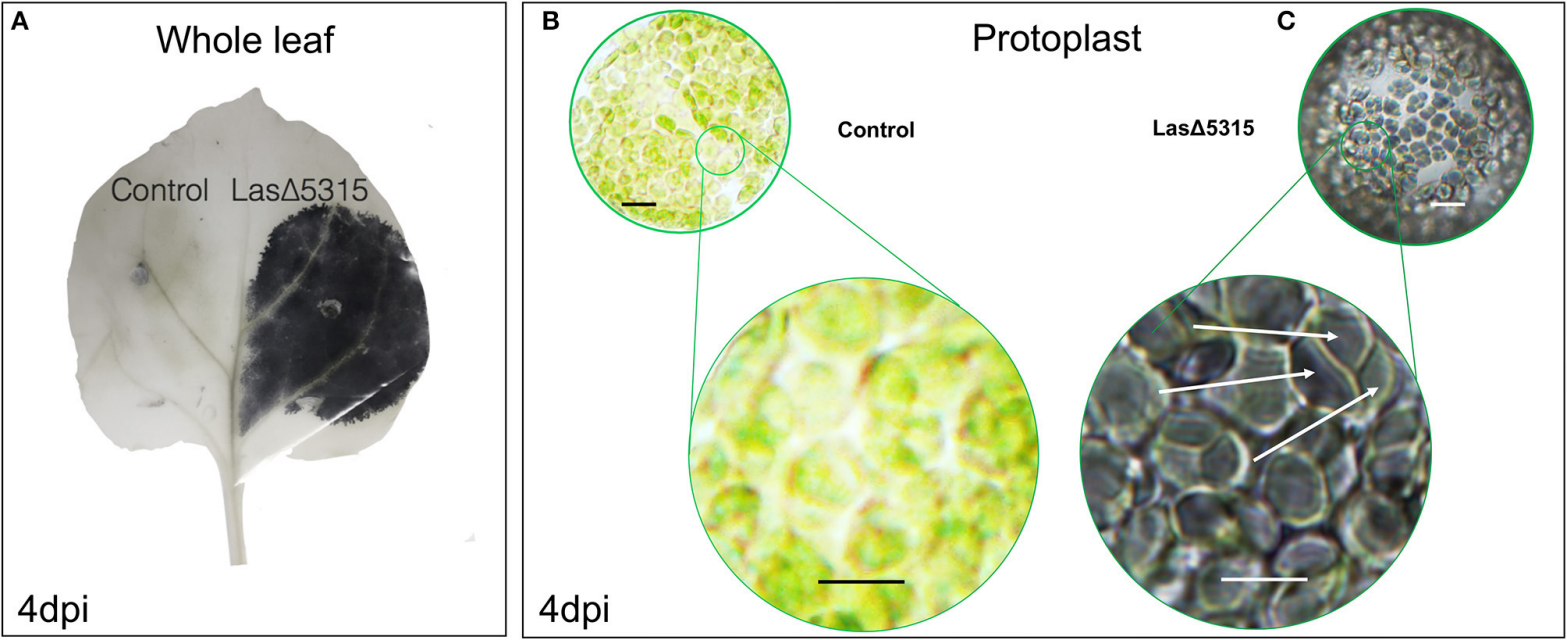
Starch accumulation in leaves expressing LasΔ5315 revealed by iodine staining. **(A)** Whole leaf infiltrated on the left expressing control vector, and on the right expressing for LasΔ5315. The LasΔ-expressing zone shows dark coloration from the iodine stain, indicating the presence of starch. **(B)** Protoplasts extracted from leaves infiltrated with a control vector (left) or a vector expressing LasΔ5315. Control appears green with little to no dark after iodine staining. **(C)** LasΔ5315 expressing tissue is stained to a dark shade by the iodine, and starch granules are visible (white arrows). Scale bar indicates 10 μM.

A major pathology associated with HLB is starch accumulation. Therefore, we investigated whether transient *Las*Δ*5315* expression in *N. benthamiana* leaves induced starch accumulation by using iodine staining for starch visualization. We observed massive starch accumulation at 4 days post-infiltration (dpi) (Figure 3A, Figure S3B). Agroinfiltration with a control vector containing GFP did not increase leaf starch content. In order to better visualize starch granules, protoplasts were isolated and similarly stained. The zone of infiltration for the control strain showed green chloroplasts in the protoplast, with no to minimal starch visible (Figure 3B). In contrast, the zone of infiltration for the LasΔ5315 strain showed dark coloration and visible starch granules (Figure 3C).

### Quantitation of starch accumulation induced by *Las*Δ*5315* expression

**Figure 4:**
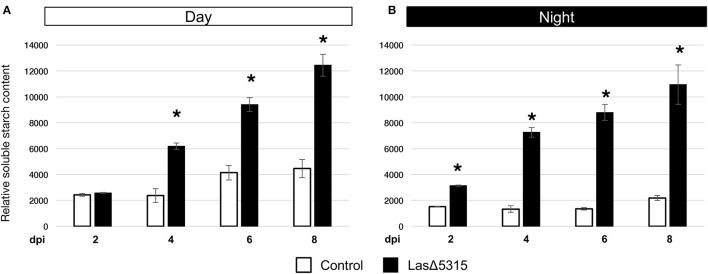
Quantitation of starch content in LasΔ5315-expressing tissues by fluorometric assay. **(A)** Relative starch content of tissues expressing a control vector (white), or LasΔ5315 (black). Samples taken after 12 h of light (daytime). **(B)** Relative starch content of tissues expressing a control vector (white), or LasΔ5315 (black). Samples taken after 12 h of dark (nighttime). Asterisks indicate significant differences between control and LasΔ5315; bars indicate standard error, the results were analyzed for significant difference with Student's *t*-test.

To quantify starch accumulation, a fluorometric assay was carried out at 2, 4, 6, and 8 dpi, both at the end (day; Figure 4A) and beginning (night; Figure 4B) of the photoperiod. The relative soluble starch content was greater in tissue expressing *Las*Δ*5315* than in tissue expressing GFP at all time points except for 2 dpi daytime. Starch concentrations increased steadily in *Las*Δ*5315*-expressing tissue throughout the time course, but stayed relatively constant in tissue expressing GFP, resulting in a 3-fold difference during the day and 5-fold difference during the night at 8 dpi. This led us to hypothesize that in tissue expressing *Las*Δ*5315*, starch synthesis enzymes were upregulated, starch degradation enzymes were downregulated, or some combination of the two.

### LasΔ5315 alters starch-related gene expression

**Figure 5:**
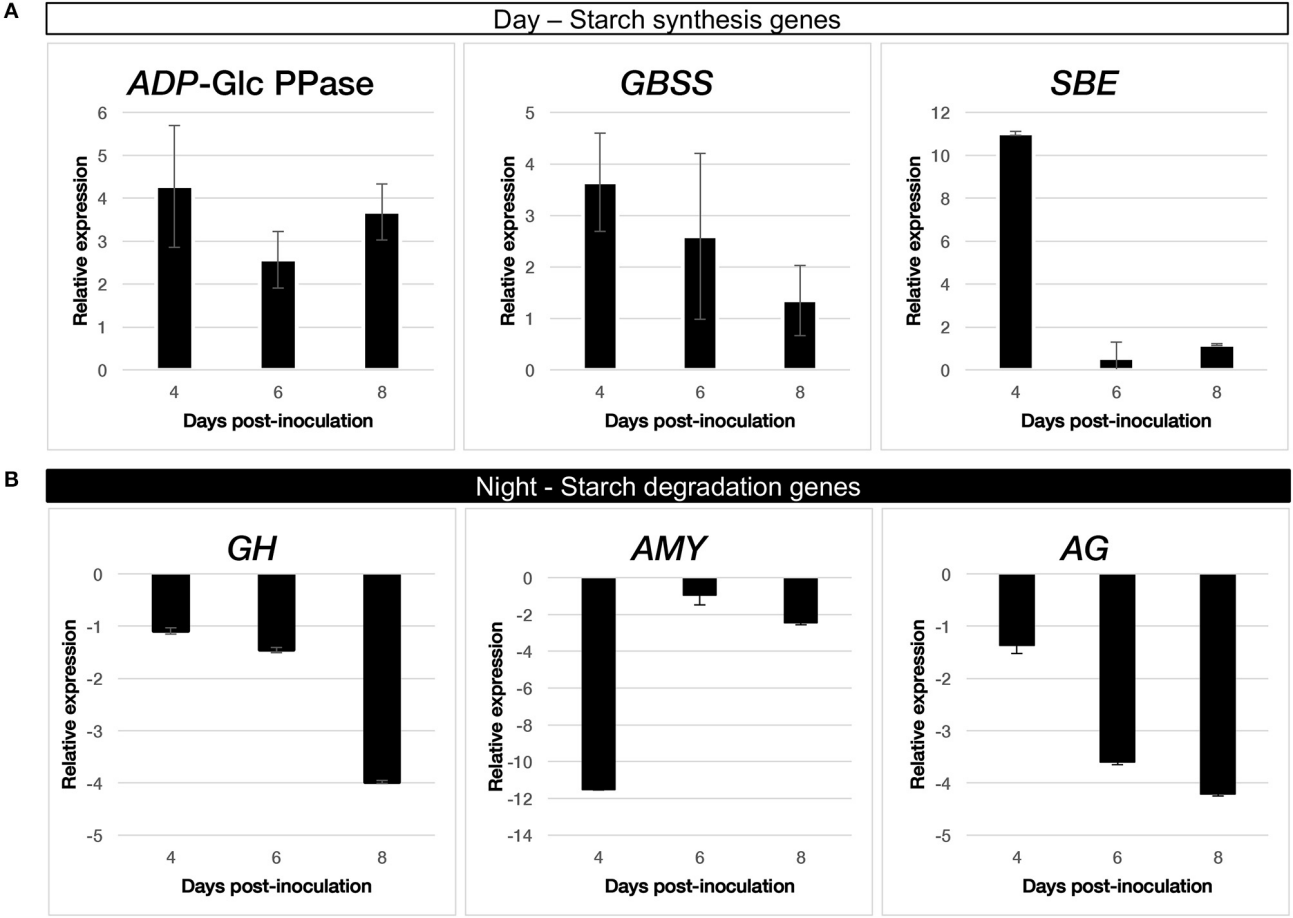
Quantification of starch-related gene transcripts by qRT-PCR in tissues expressing *Las*Δ*5315*. **(A)** starch synthesis enzymes: ADP-glucose pyrophosphorylase (*ADP*-Glc PPase), granule-bound starch synthase (*GBSS*), and starch branching enzyme (*SBE*). **(B)** starch degradation enzymes: alpha-glucosidase (*AG*), alpha-amylase (*AMY*), and glycosyl hydrolase (*GH*).

To investigate whether the starch accumulation observed in *Las*Δ*5315*-expressing tissue was due to overproduction of starch, failure to degrade starch, or combination of both, we quantified transcript levels of starch synthesis- and degradation-related genes. Since starch is accumulated during the photoperiod we quantified transcript levels of starch biosynthesis genes from samples taken near the end of the photoperiod. Since starch is broken down to serve as a source of glucose when photosynthesis is inactive, we quantified transcript levels of starch degradation genes from samples taken shortly before onset of the photoperiod by qRT-PCR. Several starch synthesis enzymes, including ADP-glucose pyrophosphorylase, granule-bound starch synthase, soluble starch synthase, and starch branching enzyme (Figure 5A) showed increased transcript levels in *Las*Δ*5315*-expressing tissue relative to controls (4, 3, and up to 10 fold difference, respectively). In contrast, transcript levels of the starch degradation enzymes alpha-glucosidase, alpha-amylase, and glycosyl hydrolase (Chiba, 1997) were down-regulated (4, 11, and 4 fold difference, respectively) (Figure 5B). These data suggest that *Las*Δ*5315* expression causes starch accumulation both by increasing starch production and decreasing starch degradation.

### *Las*Δ*5315* expression reduces chlorophyll concentration

**Figure 6:**
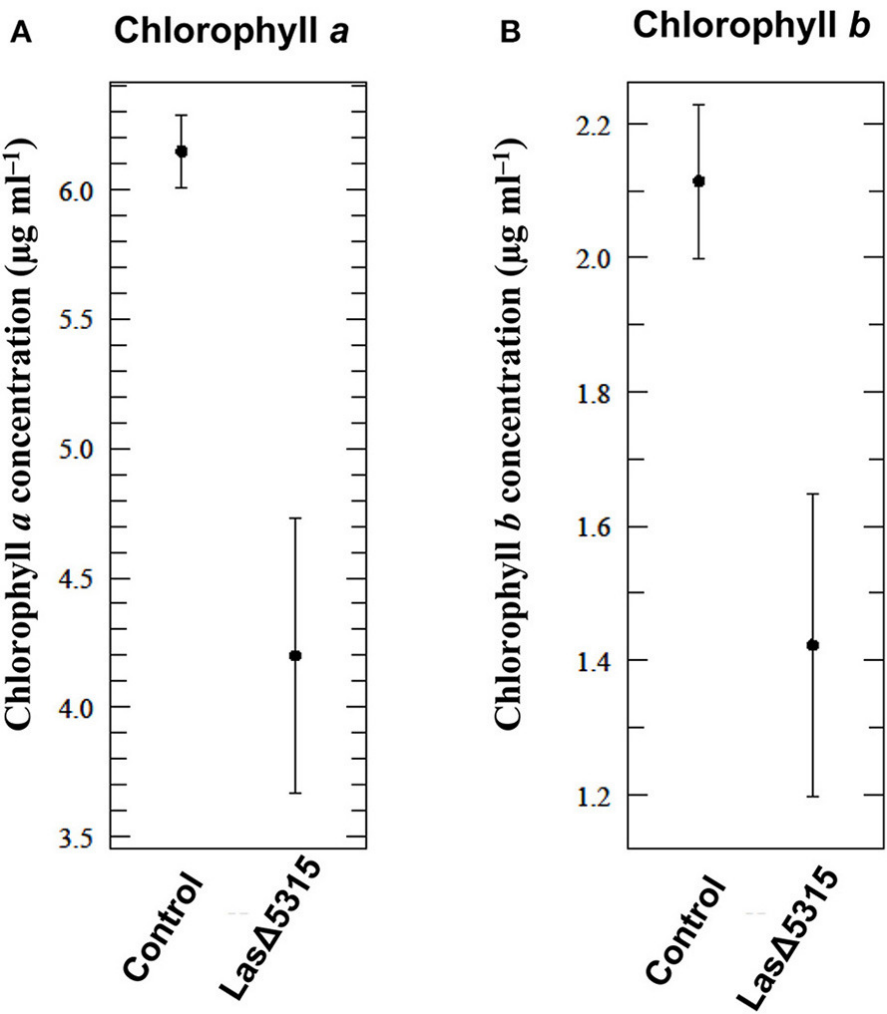
Reduction of chlorophyll content in tissues expressing *Las*Δ*5315*. Chlorophyll concentration was measured by spectrophotometric assay. **(A)** Chlorophyll *a* content was reduced in tissues expressing *Las*Δ*5315* compared to tissues expressing a control vector 4 dpi (*p* = 0.0035). **(B)** Chlorophyll *b* content was reduced in tissues expressing *Las*Δ*5315* compared to tissues expressing a control vector 4 dpi (*p* = 0.0089). The results were analyzed for significant difference with Student's *t*-test.

Starch over-accumulation and chlorosis are two of the most prominent symptoms of HLB. Chlorosis results from breakdown of the thylakoid membrane, which is thought to result from starch accumulation within the chloroplast (Schaffer et al., 1986). After observing massive starch accumulation induced by LasΔ5315, we further tested whether this phenotype was correlated with a reduction in chlorophyll, as would be expected if starch granules cause breakdown of chloroplast structures. Consistent with this hypothesis, we observed reduced concentrations of both chlorophyll *a* (Figure 6A) and chlorophyll *b* (Figure 6B) at 4 dpi in tissues expressing *Las*Δ*5315* compared to tissues expressing a control vector (*p* = 0.0035 and *p* = 0.0089, respectively). This result suggests that starch accumulation may play a role in chloroplast breakdown, and therefore be associated with chlorosis.

### *N. benthamiana* is a novel host for *Ca*. liberibacter asiaticus

**Figure 7:**
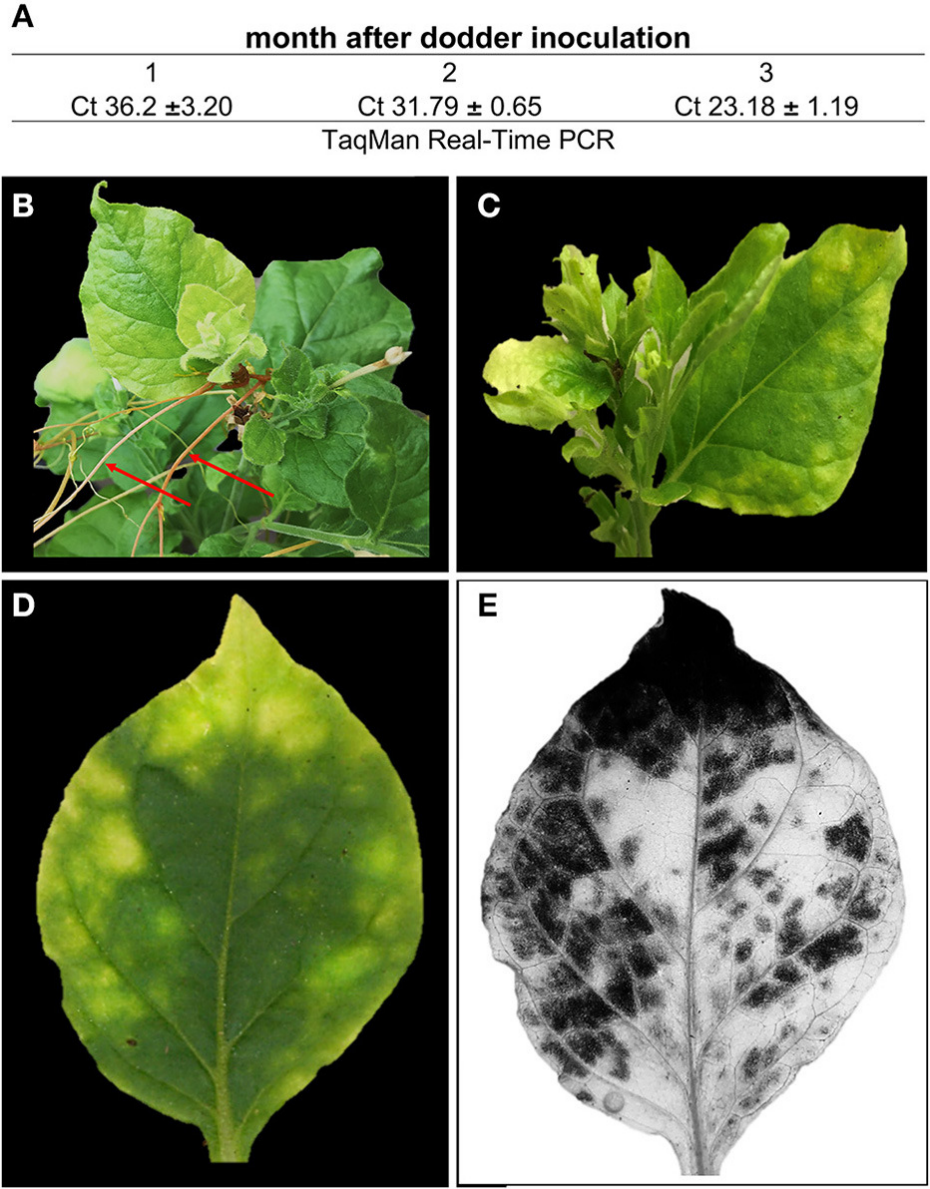
Las titer and symptoms in *N. benthamiana*. **(A)** Las titer following dodder infection. **(B)** dodder branches (red arrows) infecting *N. benthamiana*. **(C)** HLB symptoms **(D)** Las infected leaf before and **(E)** iodine stain for starch staining.

While we revealed the expression of *Las*Δ*5315* induced HLB-like symptoms in *N. benthamiana*, we confirmed that *N. benthamiana* plants were Las infected by qPCR, using primers specific for the Las 16S sequence (Li et al., 2006) (Figure 7A) and previously identified Las effector genes (data not shown) (Pitino et al., 2016). We demonstrated that *N. benthamiana* can be infected by Las via dodder transmission (Figure 7B) 2 months after inoculation, plants showed yellowing symptoms as expected (Figures 7C,E). Branches developed symptoms progressively with an uneven pattern of distribution. Like transient expression of *Las*Δ*5315*, Las infection elicited massive starch accumulation in infected tissues in *N. benthamiana* (Figure 7E).

## Discussion

HLB has devastated the Florida citrus industry, and continues to threaten citrus crops worldwide. Massive starch accumulation is thought to be the main cause of HLB symptoms. However, the molecular mechanism by which the bacterium induces starch accumulation in host plants remains unknown. Ectopic expression of Las proteins provides a convenient alternative method to study host-pathogen interactions. We previously identified Las5315mp effector, which induced cell death and callose deposition in *N. benthamiana* after transient expression (Pitino et al., 2016). Here we show that transient expression of *Las*Δ*5315* (Figure 1) causes massive starch accumulation in *N. benthamiana* leaves (Figures 3, 4), similar to that seen in Las-infected citrus and *N. benthamiana* plants. This is the first study to show a direct causal relationship between expression of a Las effector and starch accumulation. We observed that LasΔ5315:GFP was localized inside vesicles structures within the cytoplasm, many of which contained chloroplast organelles (Figures S4C–H).

Expression of *Las*Δ*5315* caused a striking phenotype, with chlorosis on the adaxial side and a water-soaking appearance on the abaxial side (Figure 2B, Figures S2A,S3B), along with reduced levels of both chlorophyll *a* and chlorophyll *b* (Figure 6). The chlorotic phenotype is characteristic of Las infection/HLB, but the water-soaking appearance on the abaxial side of the leaves expressing *Las*Δ*5315* is not similar to any known HLB symptom. However, since Las affects pathways involved in source-sink communication, including sucrose and starch metabolism (Martinelli et al., 2012), and increases potassium concentration (Nwugo et al., 2013b), it is conceivable that this Las effector could indirectly change the osmotic balance in the plant tissues. We speculate that hexoses may accumulate, leading to an increase of osmotic potential and causing water-soaking appearance of the abaxial side.

It is worth noting that rather than being a secondary result of phloem disruption, expression of this Las effector directly causes starch accumulation (Figures 3, 4). After expressing *Las*Δ*5315, N. benthamiana* tissue began to show the starch accumulation and chlorosis characteristic of HLB symptoms. These results suggest that Las5315 is a critical effector of Las, and may be directly associated with HLB symptoms. Interestingly, although starch content was consistently higher in tissue expressing *Las*Δ*5315* than in tissue expressing a control vector, greater differences were observed during the dark portion of the circadian cycle than during the photoperiod, indicating an altered regulation of starch degradation. Given that transitory starch is synthesized during the day and broken down at night, we hypothesize that failure to adequately degrade starch contributes to the observed starch accumulation phenotype more than increased synthesis.

HLB research has long been hindered by the inability to culture Las *in vitro* and by prohibitively slow citrus growth and Las transmission protocols. The use of periwinkle (*Catharanthus roseus*) as an artificial host for the bacterium has improved the situation, but genetic tools for use in periwinkle have not yet been developed. In contrast, a wide variety of genetic tools including viral expression vectors, agroinfiltration and extensive studies on plant disease resistance already are available for *N. benthamiana* (Van Ooijen et al., 2008a,b; Win et al., 2011; Du et al., 2014; El Kasmi et al., 2017). This is the first report of Las infection of the genetically tractable *N. benthamiana*. To confirm similar pathology of Las infection in *N. benthamiana* as in citrus, we analyzed starch accumulation in infected and control *N. benthamiana* plants following the normal night starch degradation phase. Upon iodine staining, infected leaves showed the same dark-colored phenotype as expression of the LasΔ5315 effector alone, indicating that Las indeed prevents starch degradation during the night, causing starch accumulation. Interestingly, when a single infected *N. benthamiana* leaf was imaged before and after iodine staining (Figures 7D,E), the darker starch stained areas co-localized with the more chlorotic parts of the leaf. In contrast, the lighter, unstained areas co-localized with greener parts of the leaf. These observations are consistent with the hypothesis that starch accumulation leads to breakdown of thylakoid and chloroplast integrity, causing chlorosis, although further testing will be necessary to prove a causal relationship.

**Figure 8:**
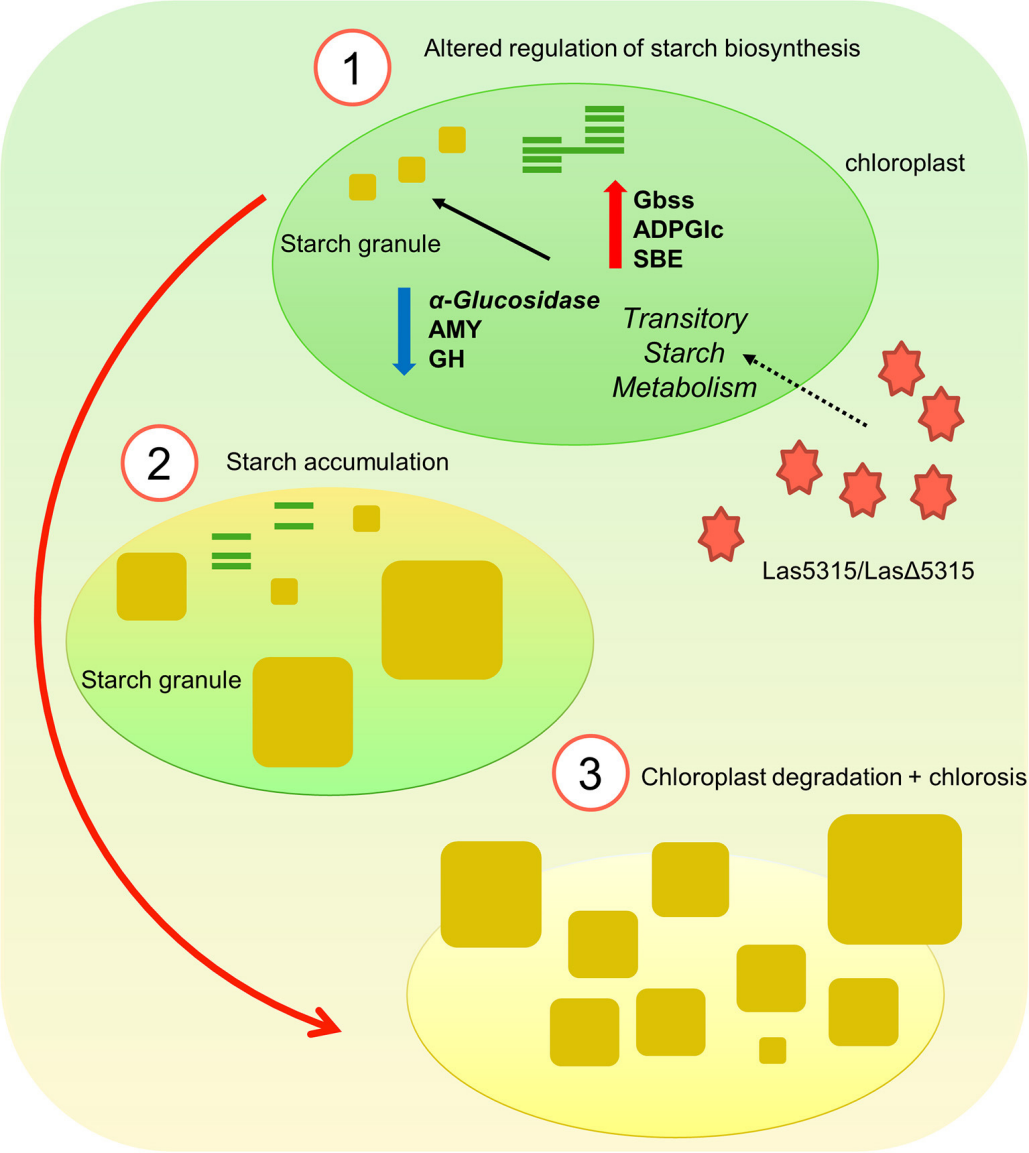
Illustration of starch accumulation and chlorosis induced by LasΔ5315. (1) LasΔ5315 modulates the transitory starch metabolism by altering starch biosynthesis. (2) Starch accumulates in the chloroplast since it fails to be degraded, possibly breaking down thylakoid membranes and thus causing chlorosis. (3) Eventually, the overabundance of starch granules causes the chloroplast membrane to rupture and the tissue is entirely chlorotic.

Las infected citrus plants accumulate large amounts of starch in the aerial tree parts (Etxeberria et al., 2009), vascular parenchyma and phloem elements (Folimonova and Achor, 2010). Several reports have shown that genes coding for ADP-glucose pyrophosphorylase, starch synthase, granule-bound starch synthase and starch debranching enzyme were up regulated and contributed to accumulation of starch in HLB-affected leaves (Ballicora et al., 2004; Kim et al., 2009; Aritua et al., 2013; Nwugo et al., 2013a), while other genes coding for key starch degradation enzymes, such as beta-amylase, glycosyl hydrolase, alpha-glucosidase, and sucrose-phosphate synthase were down-regulated (Albrecht and Bowman, 2008; Kim et al., 2009). It is likely that increased expression of starch synthesis genes and decreased expression of starch breakdown genes work synergistically to cause starch accumulation in infected tissue. Excessive starch buildup eventually causes disintegration of the chloroplast thylakoid, contributing to chlorosis, a characteristic symptom of HLB (Figure 8). To further investigate whether altered expression of starch degradation and synthesis genes was responsible for the starch accumulation observed in plants infiltrated with LasΔ5315, we analyzed starch-related genes that were affected by Las in citrus plants, both in normal diurnal and night conditions (Figure 5). Consistent with previous reports of altered transcription in Las-infected tissue, tissue expressing *Las*Δ*5315* showed increased expression of starch synthesis genes and decreased expression of starch degradation genes. Importantly, our data suggest that Las5315 effector protein may be responsible for the dysregulation of starch-related enzyme transcripts, causing starch accumulation and the consequent chlorosis phenotype.

In conclusion, here we demonstrated that the LasΔ5315 effector induced excessive starch accumulation in plant cells by modulating the transitory starch metabolism, which led to dysfunctional chloroplasts. Evaluation of starch content in Las-infected *N. benthamiana* demonstrated a failure to breakdown starch during the night. This is the first study to show a causal relationship between Las effector expression, starch accumulation, and chlorosis, indicating a potential target for novel HLB therapies. It is also the first report of Las transmission to *N. benthamiana*, which is a suitable and convenient host for studying Las due to its status as a ubiquitous model plant. Our establishment of a *N. benthamiana* Las-infection model, as well as our identification of a protein target for HLB therapies, represent important progress toward the understanding and elimination of HLB.

## Author contributions

MP and YD designed the research. MP and VA performed data collection and analysis. MP, VA, and YD interpreted the data and prepared the manuscript.

### Conflict of interest statement

The authors declare that the research was conducted in the absence of any commercial or financial relationships that could be construed as a potential conflict of interest.

## Supplementary material

Figure S1 Western blot of agroinfiltrated tissues using an antibody against LasΔ5315. Molecular mass markers (1), leaf samples expressing LasΔ5315::GFP (2), and leaf samples expressing GFP (3).

Figure S2 Visual phenotypes of leaves expressing *Las*Δ*5315*. **(A)** LasΔ5315 infiltration zone shows a chlorotic, Yellowing phenotype on the adaxial side and a water-soaking phenotype on the abaxial side. **(B)** LasΔ5315 expressing tissue is stained to a dark shade by the iodine.

Figure S3 Characteristic visible phenotypes on leaves expressing Las5315 mp and LasΔ5315, respectively. **(A)** Leaves expressing Las5315 mp show cell death as evidenced by progressive fading on the adaxial side. **(B)** Leaves expressing LasΔ5315 show a chlorotic, yellowing phenotype on the adaxial side and a water-soaking phenotype on the abaxial side. **(C)** Control leaves agroinfiltrated with a GFP vector remain healthy and green. dpi = days post-infiltration. Representative images are shown.

Figure S4 Localization of LasΔ5315:GFP in *Nicotiana benthamiana*. Epi-fluorescence and bright field micrograph of GFP and LasΔ5315 proteins. **(A,B)** GFP only; **(C,D)** Localization of LasΔ5315:GFP (green) and chloroplasts (red); **(E,F)** enlarged image showing in detail LasΔ5315:GFP localization in vesicles and chloroplasts. Scale bars indicate 20 μm.

Table S1 Primer sequences used for qRT-PCR.
