# Supplementary figures and images for: LasΔ5315 Effector Induces Extreme Starch Accumulation and Chlorosis as Ca. Liberibacter asiaticus Infection in Nicotiana benthamiana
Source: Front Plant Sci. 2018 Feb 7;9:113. doi: 10.3389/fpls.2018.00113 (PMC5808351; doi:10.3389/fpls.2018.00113)

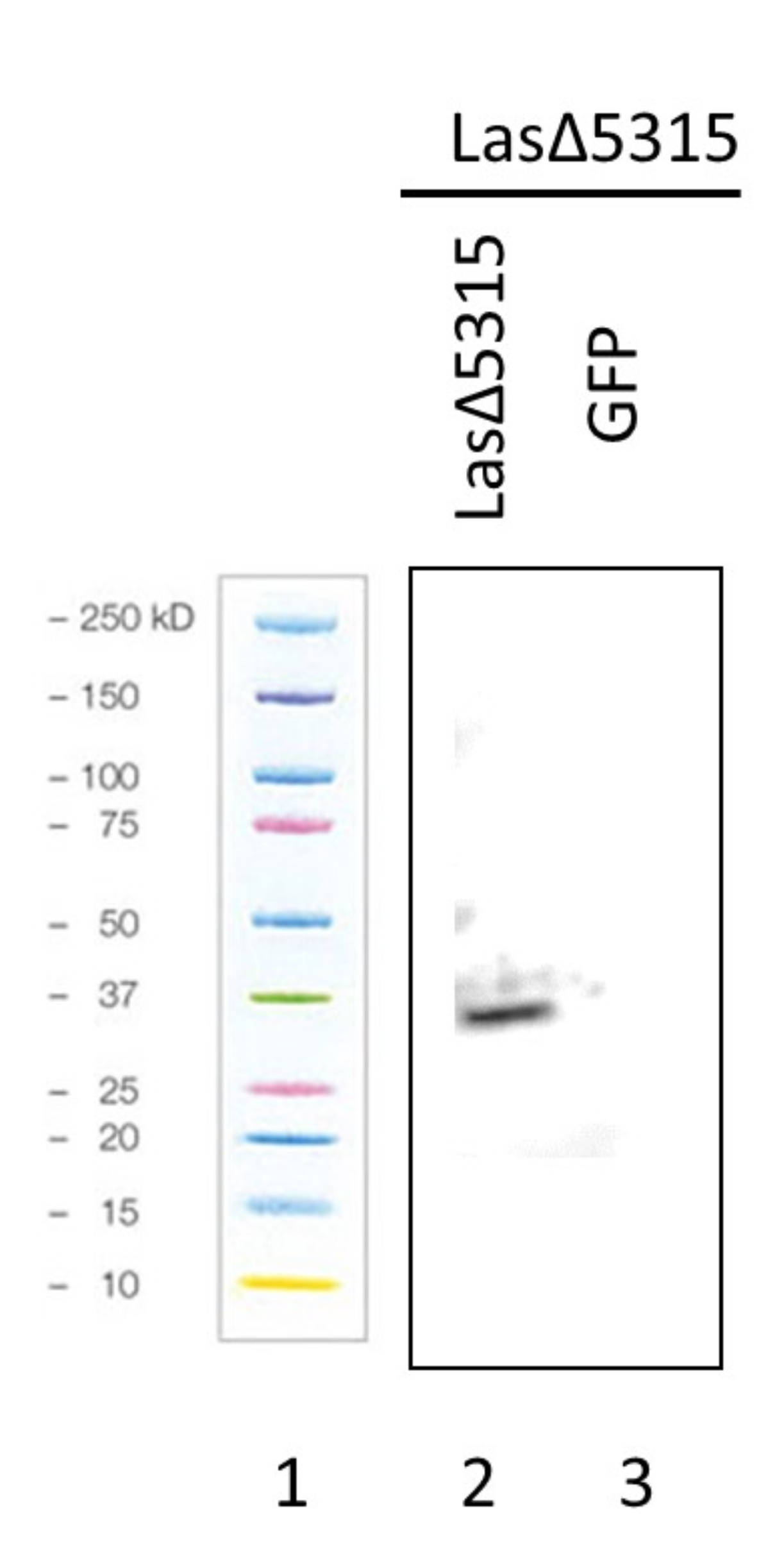

Supplement: Figure S1 — Western blot of agroinfiltrated tissues using an antibody against LasΔ5315. Molecular mass markers (1), leaf samples expressing LasΔ5315::GFP (2), and leaf samples expressing GFP (3). [file Image1.TIF]

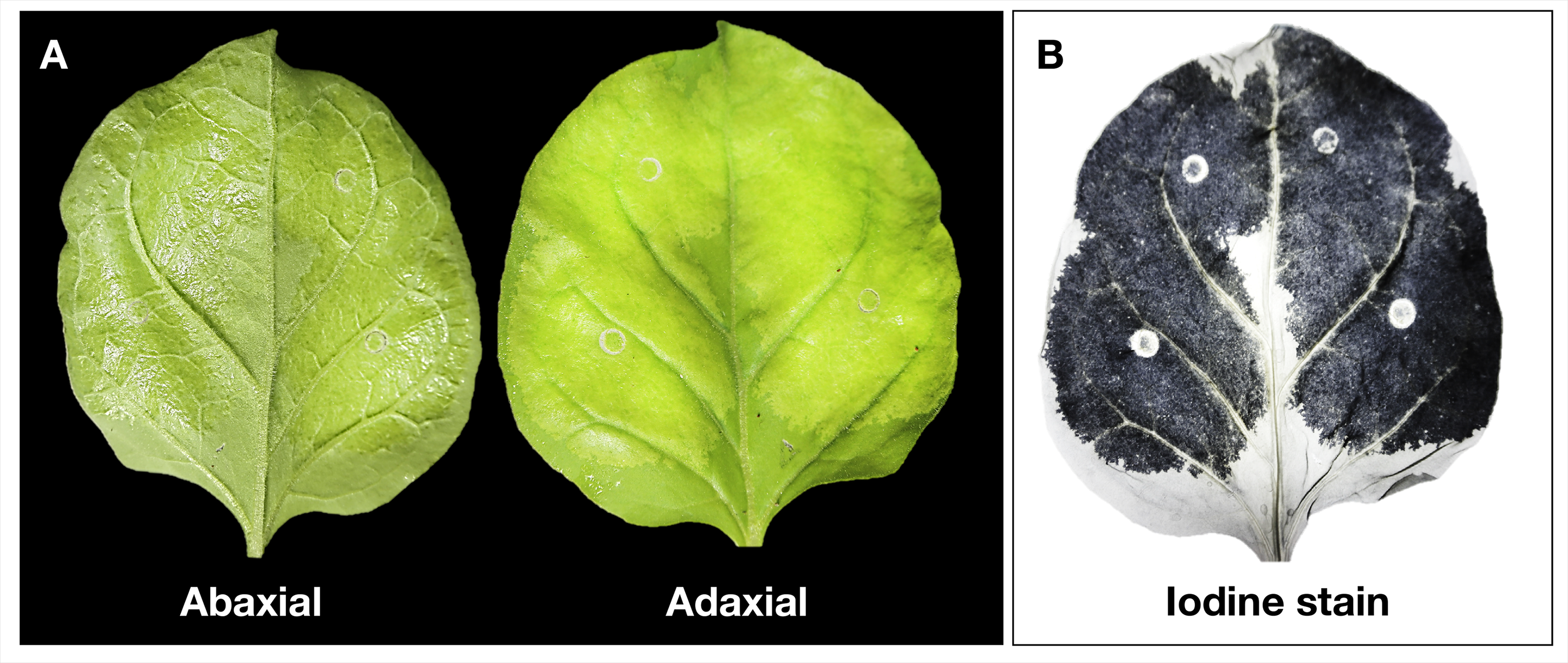

Supplement: Figure S2 — Visual phenotypes of leaves expressing LasΔ5315. (A) LasΔ5315 infiltration zone shows a chlorotic, Yellowing phenotype on the adaxial side and a water-soaking phenotype on the abaxial side. (B) LasΔ5315 expressing tissue is stained to a dark shade by the iodine. [file Image2.TIF]

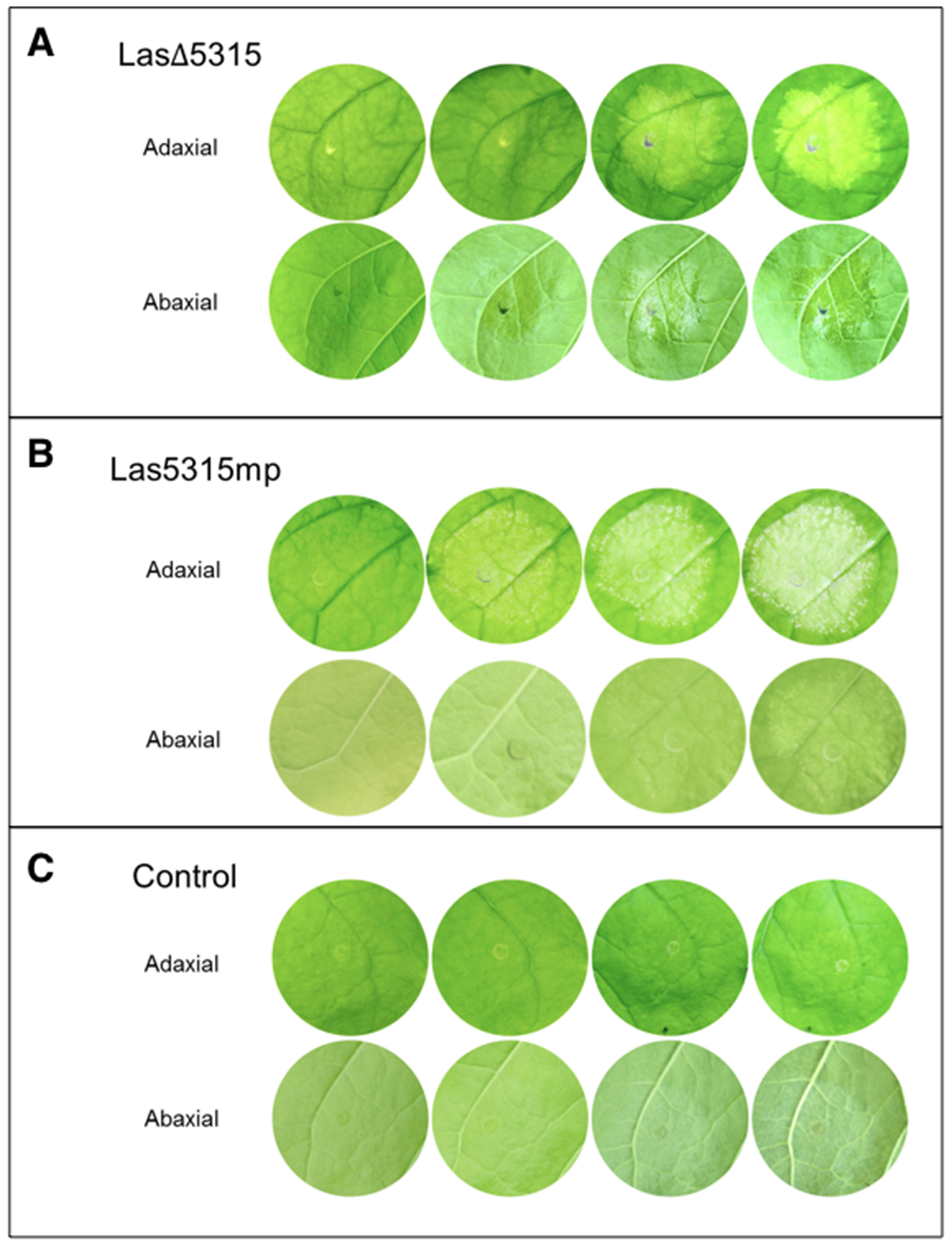

Supplement: Figure S3 — Characteristic visible phenotypes on leaves expressing Las5315 mp and LasΔ5315, respectively. (A) Leaves expressing Las5315 mp show cell death as evidenced by progressive fading on the adaxial side. (B) Leaves expressing LasΔ5315 show a chlorotic, yellowing phenotype on the adaxial side and a water-soaking phenotype on the abaxial side. (C) Control leaves agroinfiltrated with a GFP vector remain healthy and green. dpi = days post-infiltration. Representative images are shown. [file Image3.TIF]

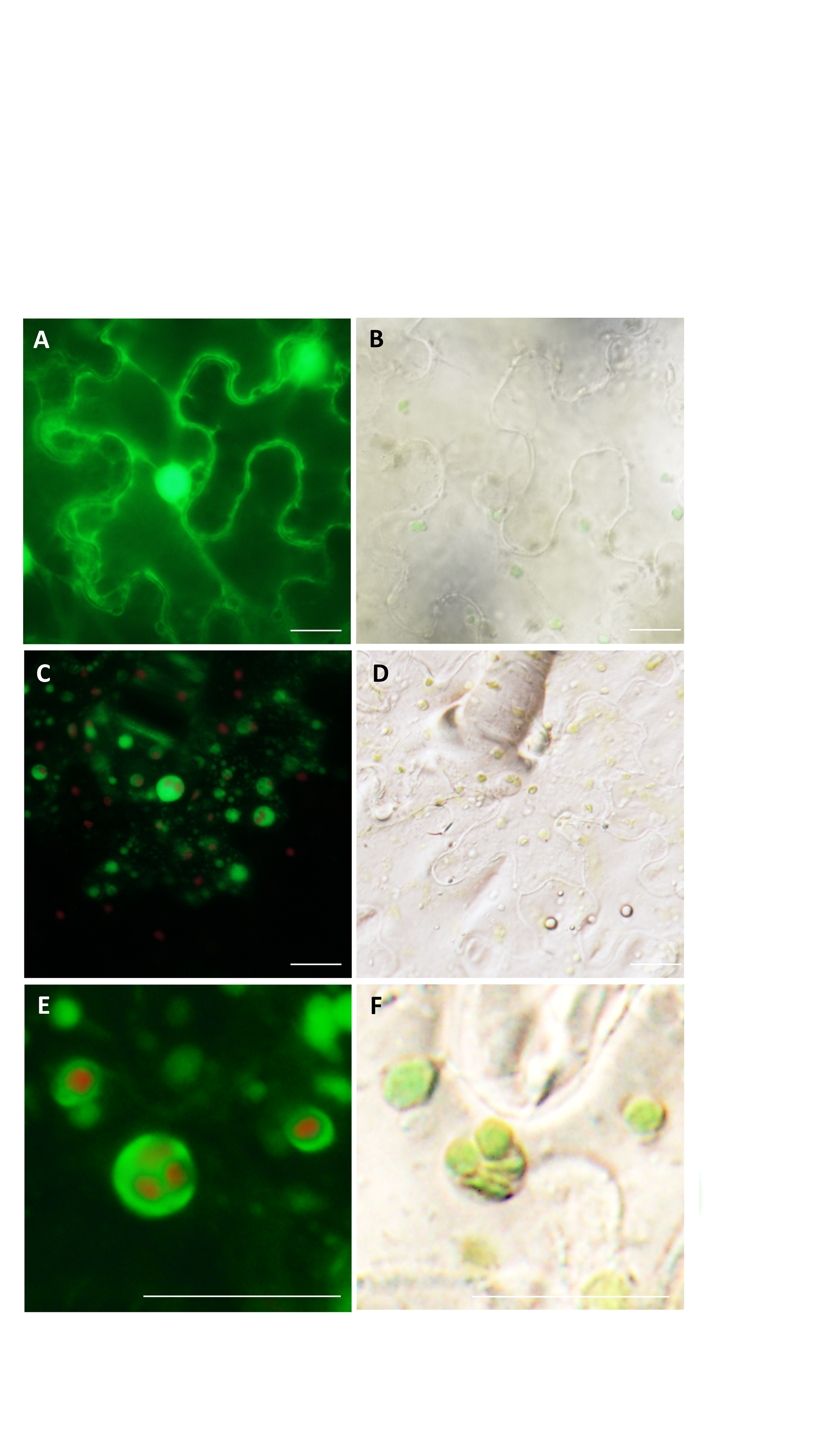

Supplement: Figure S4 — Localization of LasΔ5315:GFP in Nicotiana benthamiana. Epi-fluorescence and bright field micrograph of GFP and LasΔ5315 proteins. (A,B) GFP only; (C,D) Localization of LasΔ5315:GFP (green) and chloroplasts (red); (E,F) enlarged image showing in detail LasΔ5315:GFP localization in vesicles and chloroplasts. Scale bars indicate 20 μm. [file Image4.TIF]
