# Supplementary material for: LasΔ5315 Effector Induces Extreme Starch Accumulation and Chlorosis as Ca. Liberibacter asiaticus Infection in Nicotiana benthamiana
Source: Front Plant Sci. 2018 Feb 7;9:113. doi: 10.3389/fpls.2018.00113 (PMC5808351; doi:10.3389/fpls.2018.00113)
Supplement: Table S1 — Primer sequences used for qRT-PCR. [file Table1.DOCX]

| Primers name | Primer sequence 5’ - 3’ |
| --- | --- |
| ATTB1Sequence  ATTB2Sequence | GGGGACAAGTTTGTACAAAAAAGCAGGCTTCACC  GGGGACCACTTTGTACAAGAAAGCTGGGTC |
| ATTB1_LasΔ5315  ATTB2_LasΔ5315 | ATTB1sequence_ATGGGTTCTGCTCCTCAATCTCATG  ATTB2Sequence_AGACTGCTCCAACATTTTTCTATGG |
| impGWB405 | TCTAATCAAACAAGT  CATGGATCCTCTAGATCGAAC |
| Alpha-amylase | Atggaacctggcagaaatttgt  ggcagaatcccaataagaacatg |
| Alpha-glucosidase | TAACGTCGATGGACAACCAA  AAGGCAATTCCAGTCCATGA |
| ADP-glucose pyrophosphorylase | Gctcccttgaatcgtcacat  tcttggcatcctcaaaaacc |
| EF1 | TGAGATGCACCACGAAGCTC  CCAACATTGTCACCAGGAAGTG |
| Glycosyl hydrolase | GAAAAACCATTGACTGCTTGC  GCTCTCAGCCACCATTACAAA |
| Granule-bound starch synthase | Gctcaaatgtctgcagtgga  cggatacctgtttgctggat |
| Starch branching enzyme | Gcagaacggcagtggtctat  acatgaaactccgccaagac |

Supplementary Table 1. Primer sequences used for qRT-PCR.
